# The role of micro, small and medium enterprises (MSMEs) to the sustainable development of sub-Saharan Africa and its challenges: a systematic review of evidence from Ethiopia

**DOI:** 10.1186/s13731-022-00221-8

**Published:** 2022-02-21

**Authors:** Ebrahim Endris, Andualem Kassegn

**Affiliations:** Department of Agricultural Economics, Woldia University, Woldia, Ethiopia

**Keywords:** Employment, Enterprises, Ethiopia, Finance, Sustainable development

## Abstract

Micro, small and medium-sized enterprises (MSMEs) have a potential impact on achieving many of the sustainable development goals much greater than their size. This review aimed to investigate existing literature on the contribution of MSMEs to the sustainable development of Ethiopia and its challenges. The review provides a comprehensive and systematic summary of evidence and provides future research directions. A systematic review methodology was adopted through reviewing the available literature comprehensively including research articles, policy documents, and reports over the period 2011–2021 from ScienceDirect, Google Scholar, ECONBIZ, IJSTOR, EBSCO, Web of Science, and Scopus databases. A search on these databases and grey literature returned 1270 articles; 87 papers were included in this review following screening of aticles using pre-determined criteria. The paper found that MSMEs significantly contributed to the sustainable development goals of Ethiopia through creating employment, alleviating poverty, and improving their living standards. However, the review has identified access to finance, access to electricity, and trade regulation are the major constraints for the development of the sector. The review outlines key policy implications to develop a comprehensive policy that alleviates the existing challenges of the sector and calls for further MSMEs impact evaluation research.

## Introduction

Growth in the working age population is expected to be even more rapid, increasing by 265.8% in Africa and by 306.6% in sub-Saharan Africa, compared to 28.3% globally (Bhorat & Oosthuizen, 2020). Consequently, unemployment is a colossal problem in sub-Saharan Africa (Dey, 2012). Entrepreneurship can be a cure for Africa’s problems such as unemployment, inequality, low productivity, disconnect from global value chains, etc. (Devine & Kiggundu, 2016). The General Assembly adopted resolution 71/221 recognizes the important contribution entrepreneurship to sustainable development by creating jobs, driving economic growth and innovation, improving social conditions, and addressing social and environmental challenges (UN, 2018). Hence, investment in entrepreneurial ventures can contribute immensely to economic growth and job creation (Arko-Achemfuor, 2017) and thus jobs provide income, which improves living standards and consumption possibilities (IFC, 2013). Micro, small and medium-sized enterprises (MSMEs) are a major source of growth, innovation and jobs and their potential impact on achieving many of the sustainable development goals is much greater than their size (ITC, 2019). Therefore, there is a great interest of young people to start a business and many of them are willing to undertake risks and challenges of entrepreneurship (Papulová & Papula, 2015).

Sustainable development goals (SDGs) in Africa emphasize on labor-intensive sectors (SDG 8.2), increase small-scale enterprises’ access to affordable credit in support of decent job creation and entrepreneurship (SDG 8.3 and 9.3) (Brixiová et al., 2020). The informal sector (nonfarm) has been a growing source of employment for a large section of the African youth, but also for older workers trying to seize entrepreneurial opportunities. Its contribution to GDP and poverty reduction has been substantial, and it has become a major point of entry into the labor market (AFDB, 2019). Small and medium-sized enterprises (SMEs) make crucial contributions to job creation and income generation. The promotion of SMEs has been a key area of intervention in recent years in view of the major employment challenges (ILO, 2015). For that reason, the employment share of the self-employed in low-income countries is almost five times (54%) the share in high-income countries (11%), and the employment share of micro-enterprises (2–9 employees) also much higher (ILO, 2019b). Small and medium enterprises have embraced technological innovations in creating new opportunities as well as expanding their businesses. In particular, high mobile phone penetration has brought opportunities to SMEs in rural and urban areas of Africa (Amankwah-Amoah et al., 2018).

Ethiopia most important development priorities were job creation for the increasing supply of labor force which contributed in reducing poverty (NPC, 2016; WBG, 2018). Hence, the implementation of the micro and small enterprises (MSEs) development strategies given undue role to achieve these objectives (NPC, 2016). The revised MSE strategy focus on enhancing the competitiveness of MSEs, ensuring continued rural development through sustainable growth of MSEs, and making the subsector a foundation for industrial development (FMSEDA, 2011). During Growth and Transformation Plan (GTP) I implementation period (2010/2011–2014/2015), construction sector was largest over other sector which accounts about 36.2%, followed by services with 20.8%, trade with 15.2%, manufacturing with 14.7% and urban agriculture accounts 13.1% employment through MSEs (EEA, 2015).

Establishment of MSEs strategy by itself cannot alleviate the problems facing MSEs and improve the development of the sectors (Hunegnaw, 2019). The ability of the firm to operate for longer time depends up on a proper tradeoff between management of investment in long-term and short-term funds (Dinku, 2013). The more rapid growth of small firms in Ethiopia is offset by a very high rate of firm failures (Page & Söderbom, 2015), this risk of business failure is high during the first 2–4 years of business operation (Woldehanna et al., 2018). Given the implication of MSMEs to the national development goals and it is a key development policy, there is little evidence that explore its role and prevailing challenges in a broader context. Hence, this review article aimed to provide an exploratory insight on the contribution of MSMEs in achieving sustainable development of Ethiopia and identify the prevailing challenges. The review contributes to the existing literature by providing evidence for these specific questions. (1) What is the role of MSMEs in attaining sustainable development goals of sub-Saharan Africa specifically Ethiopia? (2) What are the challenges hindering the development MSMEs in the country? This literature review identifies the specific research gaps uniquely relevant for future researches and policy direction for the development of the sector.

## Review methodology

The review adopted a systematic literature review method, which offers an explicit, trustworthy, and reproducible method to minimize bias, thus providing more reliable findings for the evaluation and interpretation of previous research relevant to a particular field (Sniazhko & Muralidharan, 2019). The review based on extensive overview of relevant literature (research articles, policy documents, and reports) following a systematic review approach utilizing PRISMA guidelines (Liberati et al., 2009).

### Literature search

The review retrieved from international databases using keywords identified. The literature search was conducted in ScienceDirect, ECONBIZ, IJSTOR, Google Scholar, EBSCO, Web of Science and Scopus databases that provides large collection of articles. The literature search was done using the following keywords: ((“micro enterprise” OR “small enterprise” OR “enterprise” OR “sustainable development”) AND “Ethiopia” OR “Africa”) in the citation information, keywords and abstracts. Moreover, we conducted a snowball search by examining the reference lists of included studies to include additional relevant studies that might have been missed for a variety of reasons. In addition, national university research repository used to search relevant thesis and dissertation to obtain a comprehensive set of evidence. The review used secondary data extracted from international organization databases such as World Bank, IFC, ILO, and NBE to support the review with empirical evidences. These databases are recognized as the key sources for retrieving relevant, up-to-date articles in socio-economic field, and are commonly used by other scholars to conduct systematic review (Sniazhko & Muralidharan, 2019). The preliminary searches within the databases using the abovementioned keywords identified 1270 records.

### Study identification and the screening and selection process

The two fundamental components in a systematic literature review are (i) deciding on the inclusion and exclusion criteria of studies, and (ii) assessing the quality of the studies to be included (Čablová et al., 2017). The preliminary extensive list of identified articles was narrowed down to specifically relevant literature through inclusion and exclusion criteria. The articles retrieved from online database searches and different sources were collected in to Endnote Library. The articles identified in stage one was examined thoroughly to exclude the duplicated articles of the same titles that were available in multiple search databases. For the initial search, we set three inclusion criteria: (1) any literature that include at least one of key terms or words (2) literature written in English language, and (3) conducted over the last 10 years (2011–2021). In addition, filter criteria were applied to reduce the number of articles based on (1) articles published before 2011, (2) editorial comments, book reviews, and review articles, and (3) any literature out of the scope of this review were excluded. Then, repetitive articles, and articles not related to the subject using the inclusion criteria were excluded. By applying these inclusion and exclusion criterions, the search generated 210 records.

**Fig. 1:**
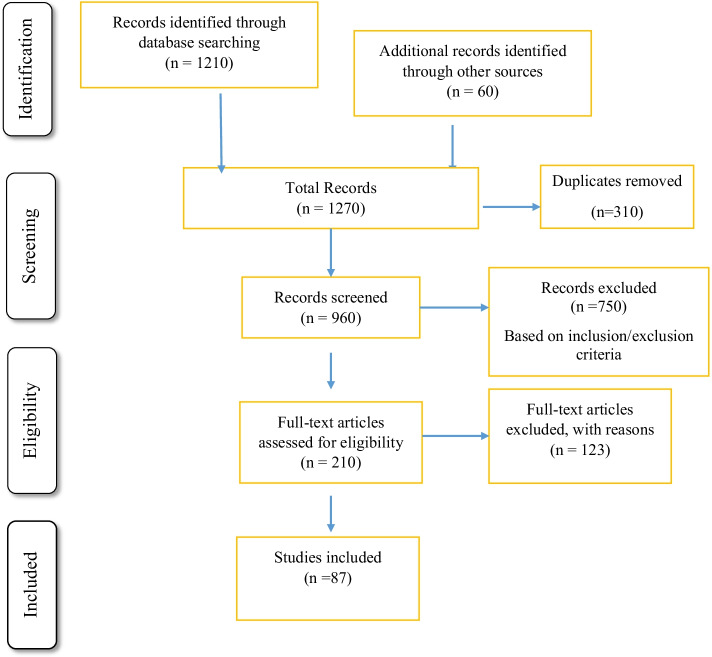
Study selection process (PISMA flow diagram)

Our search identified 1270 retrieved records, which were reduced to 960 after removing duplicates. Two of the researchers (E.E and A.K.) who used the above criteria to determine paper eligibility to be included in the study, reviewed titles and abstracts independently. From theses, 210 articles were identified eligible for full-text review after screening title and abstracts for final inclusion. There are 123 articles excluded because no empirical evidence relevant for this review. Any disagreements regarding the exclusion of an article were resolved through a discussion among the authors, through multiple round reading when necessary to achieve consensus. The details of procedures presented in the PRISMA flow diagram (Fig. 1), and 87 studies finally reviewed comprehensively in an attempt to identify the findings within the articles. The findings in the articles synthesized qualitatively to provide answers for review questions.

## Results and discussion

### Implication of MSMEs towards sub-Saharan Africa sustainable development

Entrepreneurial activity is crucial to the achievement of multiple SDGs, including SDG 1: “End poverty in all its forms everywhere”; SDG 8: “Promote inclusive and sustainable economic growth, employment and decent work for all”; SDG 10: “Reduce inequality within and among countries” (Bosma et al., 2020). The United Nations’ SDG 8 sets out a global consensus that business enterprises should aim for sustained, inclusive, and sustainable economic growth and also ensure decent work and living environments for all (Lin & Koh, 2019). Small and medium-sized enterprises play a key role in job creation, providing two-thirds of all formal jobs in developing countries and 80% in low-income countries. The sustained success of SMEs depends on local conditions, such as public services, good corporate law and access to finance (EDFI, 2016). In addition, MSEs provides a substantial collective contribution to the national economy (White, 2018), contribute more than 50% of most African GDP and an average of 60% of employment (Muiruri, 2017). It employs the vast majority of any local labor force and has an integral role in any sustainable growth trajectory and it is ‘the missing link’ for inclusive growth (ITC, 2018). Although African SMEs generate about 80% of new jobs, they also account for most lost jobs (ILO, 2019a). Micro enterprises in Ethiopia account the greatest share of employment from developing countries (IFC, 2013).

Investing in SMEs can contribute to 60% of the targets established in the SDGs and about $1 trillion additional SME investment help developing countries reach the SDGs. Small and medium enterprise contribute to 83% of SDG 8 (Promote sustained, inclusive and sustainable economic growth, full and productive employment and decent work for all) targets, and 88% of SDG 9 (Build resilient infrastructure, promote inclusive and sustainable industrialization and foster innovation) targets (ITC, 2019). Hence, financial stability in sub-Saharan Africa enhances entrepreneurial development which improve economic growth and accelerated achievement of SDGs (Babajide et al., 2020). There is a large concentration of enterprises in sub-Saharan Africa, 44 million MSMEs, of which 97% are micro-enterprises, of which the largest share (37 million MSMEs) accounted by Nigeria enterprise (IFC, 2017).

Even though, the contribution of MSEs to total employment and gross job flows were underestimated (Li & Rama, 2015), it contributed to economic growth through their operational activities, via job creation in Nigeria economy (Matthew et al., 2020) and micro-enterprises alone account for a staggering 97% of manufacturing sector employment in Ethiopia (Li & Rama, 2015). Entrepreneurship and new venture creation in South Africa emphasize on employment opportunities for MSMEs employees, and the social dimensions of poverty reduction approaches are broader than these economic imperatives (Rambe & Mosweunyane, 2017). Small-scale enterprises employment has absorbed over 49% of the increase in the labor force in five countries of sub-Saharan Africa (Botswana, Kenya, Malawi, Swaziland and Zimbabwe). Similarly, about 80% of employment growth in Tanzania accounted by informal enterprises (Diao et al., 2018).

#### Problems in the development of enterprise in sub-Saharan Africa

Entrepreneurial activity in low-income countries dominated by individuals who are forced into starting their own business due to a lack of employment and typically not highly productive (Doran et al., 2018). The main challenges constrained SMEs contribution to local economic development of developing countries are lack of finance, lack of business skills, poor market access, and lack of operating space (Gebreyesus & Adewale, 2015). The contribution of MSMEs to sustainable development is constrained by unfavorable business environments, inadequate access to finance and high levels of informality (ITC, 2019). The challenges such as lack of access to finance, weak entrepreneurial attitudes, government policies, regulations and practices for entrepreneurs, and training are main constraints to SME development in sub-Saharan Africa (Achtenhagen & Brundin, 2016; Herrington & Coduras, 2019; IFC, 2011). Due to this, the core focus of the owner of MSMEs are personal financial survival rather than on growing and developing the business, which affected the success of small businesses. In these circumstances, profit made in the business is often spent on personal expenses rather than being reinvested into the business (IFC, 2020b).

Accessing finance for entrepreneurship development in Africa is still continuing and new challenges to MSMEs (Atiase et al., 2017; Beck & Cull, 2014). Access to credit currently fails to support entrepreneurship development in Africa (Atiase et al., 2017; Wang, 2016), and SMEs have limited access to finance even though banks have sufficient liquidity (Brixiová et al., 2020). Most financial institutions undermine smaller enterprises and instead focus on big businesses that can provide the required collateral for their loans (Atiase et al., 2017). Difficulty to obtain formal credit were due to small capital of MSEs below critical collateral value (lack tangible assets as collateral) (Jin & Zhang, 2019), high risk premiums, and higher transaction cost to banks, as SMEs loan size are generally small (Quartey et al., 2017). Sub-Saharan Africa have low financial inclusion index (Ofori-Abebrese et al., 2020). Hence, access to finance remains the largest obstacle for SMEs in the region and 75% of enterprises were financed by internal funds and other 10% used traditional banking loans (Leke & Signé, 2020). For example, 79% of informal businesses have never obtained loan, and only 21% utilized bank loan in South Africa. From this, only 19% of formal businesses used a bank loan to start their business (IFC, 2020b). In Tanzania, only 30% of MSMEs had access to financial services (Ishengoma, 2018). A large number of enterprises in sub-Saharan Africa are occasional enterprises that function for a limited period of the year. For instance, lack of profitability and a lack of finance the most important reasons for enterprise exit in Uganda (Nagler & Naudé, 2016). Furthermore, lack of finance and harsh business environment tends to constrain the growth of MSMEs in Uganda (Lakuma et al., 2019), access to finance is still a hurdle to MSMEs establishment in Lesotho (Khoase & Govender, 2013), and access to both debt and equity markets also affected micro-enterprises in South Africa (Fatoki, 2017).

The limitation of finance has an inhibiting effect on the growth of African firms (Fowowe, 2017). For example, financially constrained firms have 6.6% lower marginal revenue product of capital relative to unconstrained firms. Moreover, constrained firms are also more inefficient and less productive relative to unconstrained firms in sub-Saharan Africa. Constrained firms are 15% less efficient due to borrowing constraints compared to unconstrained firms (Amos & Zanhouo, 2019). For instance, SME access to bank finance can further increase the contribution of SMEs to the Ghanaian economy and increase their chances of survival and success through exports (Abor et al., 2014).

The low performances in sub-Saharan Africa attributed exclusively to factors outside firms, such as poor infrastructure and unfavorable governance (Mano et al., 2012). The risks faced by entrepreneurs in Nigeria SMEs arose from the increasing complexity and sophistication of the industrial sector and increasing macroeconomic instability (Ejembi & Ogiji, 2017). The operational environment of SMEs strongly indicate that their productivity is constrained by lack of adequate infrastructure as well as inefficient institutions in Nigeria (Effiom & Edet, 2020). The lack of business infrastructure hampers MSMEs’ ability to scale and grow in South African, lack of equipment as the second largest challenge at startup (IFC, 2020b), and limited awareness of government program (Fatoki, 2017). The results further indicate that the majority of MSMEs had no access to public infrastructure, i.e., only 16% and 28% of MSMEs had access to electricity from the national grid and water from the public or municipal sources, respectively (Ishengoma, 2018).

#### The impact of COVID-19 on sub-Saharan Africa MSMEs

COVID-19 lockdowns and social distancing measures influenced the MSMEs operation. Finding from study carried out in 132 countries revealed that two-thirds of micro and small firms reported that COVID-19 has affected their business operations and one-fifth of SMEs confirmed they face risks of closing down permanently within 3 months (ITC, 2020). The COVID-19 outbreak has posed great challenges for the survival and growth of SMEs (Guo et al., 2020). The upheaval caused by the spread of COVID-19 have a devastating effect on small businesses. Moreover, the economic fallout from this pandemic get worse for small businesses and their employees (Liguori & Pittz, 2020). The feature of MSMEs such as more labor-intensive activity hurt during COVID-19 lockdowns, limited reserves and lack of collateral for new credit lines are key factors which make SMES highly vulnerable to the impact of COVID-19 pandemic (COMESA, 2020).

The study on 367 agri-food MSMEs from 17 low and middle income countries revealed that 94.3% of firm’s operations had been impacted by the pandemic, primarily through decreased sales as well as lower access to inputs and financing amid limited financial reserves. Moreover, 84% firms reported changing their production volume as a result of the pandemic; of these, about 13% reported stopping production and about 82% reported decreasing production (Nordhagen et al., 2021). The pandemic has severely affected about 37 million microenterprise and 28,000 SMEs due their lack of adequate cash buffers and access to finance. About 25 million micro-enterprises operating in tourism, hospitality, entertainment, and trade had to close or face significantly reduced operating hours (IFC, 2020a). Therefore, COVID-19 is a substantial threat to the attainment of SDGs 1, 2, 3 and 8 in Nigeria (Ogisi & Begho, 2021). The pandemic severely hurt financial health of MSMEs in sub-Saharan Africa via reduced profit, turnover decrease, and liquidity crunch. The percentage of MSMEs that suffered due to the pandemic is presented (Table 1) for some countries for which data were obtained.

**Table 1:** Impact of COVID-19 on MSMEs financial performance

Country	% MSMEs impacted by COVID-19 (%)	% of enterprises profit/turnover/decreased	% of business reported cash flow problem (%)
Guinea	97	87% (on average by 38% profit)	85
Serra Leone	96	81% (on average by 39% profit)	71
Cote d’Ivoire	93	78% (on average by 34% turnover)	75
Uganda	100	89% (on average by 49% turnover)	73
Kenya	67	69% (on average by 40% turnover)	67

Source: summarized from IFC—COVID-19 business impact series (2020)

### Role of MSMEs development in Ethiopia

Micro and small enterprise development is the primary strategy of GTP II to expand employment and reducing poverty particularly focusing on women and youths (NPC, 2016). The government of Ethiopia proposed MSEs as means of creating employment to millions of youths and achieving sustainable development goals. Hence, there is policy support leads SMEs generating more employment compare to large firms (Ashenafi, 2014). Therefore, the contribution of MSEs to employment creation is much higher (99%) than that of medium and large enterprises (1%) (Abera et al., 2019). A sizable number of people are employed in small-scale tourism enterprises with a decent average monthly income that can improve their living standard in Hawassa City (Tamene & Wondirad, 2019). Similarly, MSEs program had led to positive outcomes on the income and livelihood of beneficiaries in Bahir Dar City (Melese, 2017). With respect to sector contribution, manufacturing and construction enterprise ranked first and second, respectively, in creating job opportunities for job seekers in Kolfe-Keranio Sub-City, Addis Ababa (Tafa, 2019). Manufacturing and urban agriculture sector provide huge contribution in reducing food insecurity of operators in Mecha district (Yimesgen, 2019).

**Fig. 2:**
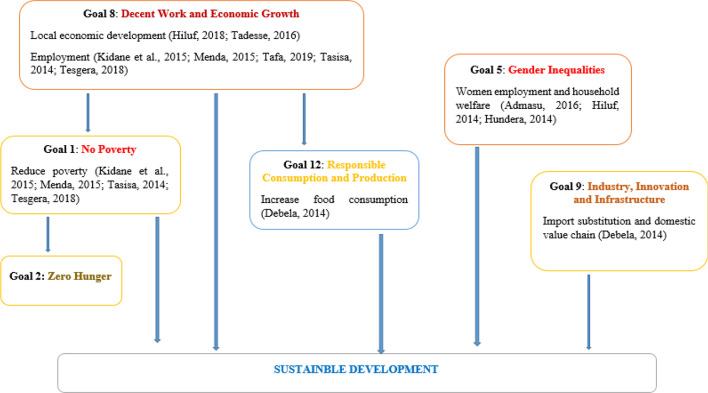
Framework on MSMEs contribution to sustainable development. Source: own sketch based on literature review

The conceptual framework on the role MSMEs towards SDGs is presented in Fig. 2. The framework showed that MSMEs has positive implication in meeting SDG 1, 2, 5, 8, 9, and 12 (Bosma et al., 2020; ITC, 2019; Lin & Koh, 2019).

**Fig. 3:**
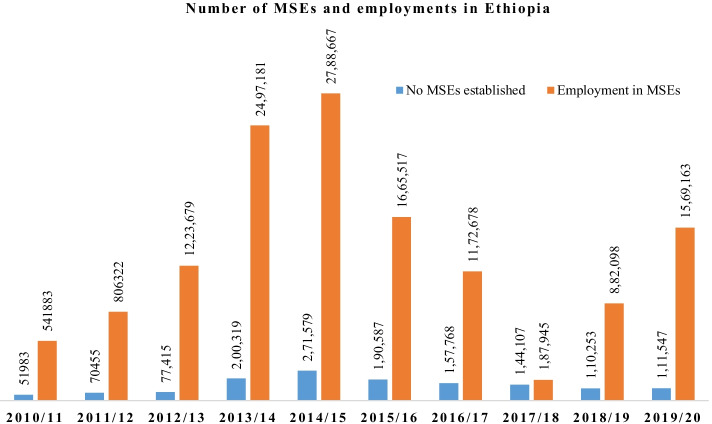
Number of enterprise and employment created in Ethiopia. Source: authors calculation from NBE (2020)

Employment in micro-enterprises leveled as high in terms of its extent of importance to poverty reduction (Kidane et al., 2015). It have played a positive role in women’s livelihood by creating employment opportunity for those who are in need of job and with low level of income, empowered them socially and economically (Admasu, 2016; Menda, 2015). In addition, entrepreneurs have created job opportunities to others while also contributing to local economy and communities through income tax payment. It provides annual average of minimum 5–7 and maximum 17–23 employment opportunities in the last 5 years. The annual average income of the enterprises was at the minimum ranging between 30,000–50,000 Birr and maximum ranging between 141,001–200,000 Birr (Hiluf, 2018). The distribution of MSEs established and number of employment opportunities in the enterprise varying across years. Based on Fig. 3, the number of enterprise and employment in MSEs was largest in 2014/2015.

### Challenges of MSME in Ethiopia

#### Financing MSMEs in Ethiopia

**Fig. 4:**
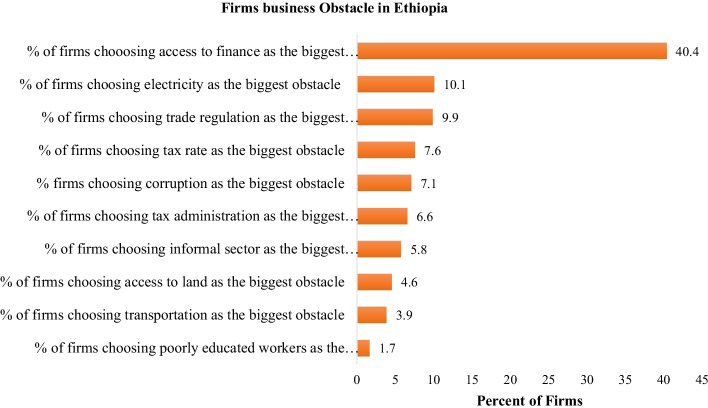
Financial challenges across enterprise size. Source: Author calculation from World Bank enterprise survey (2015)

**Fig. 5:**
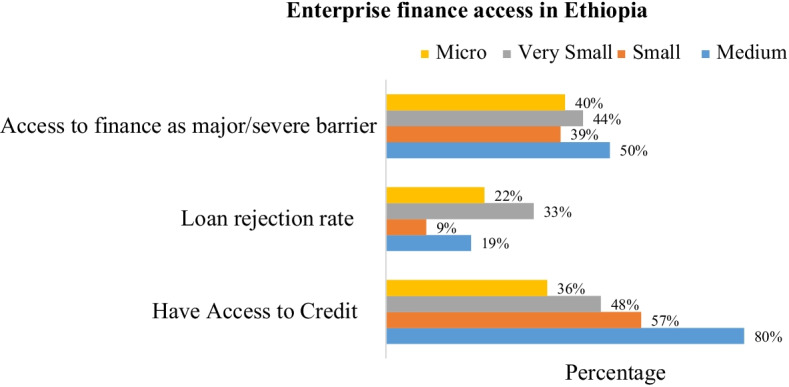
Financial challenges across enterprise size. Source: author calculation from IFC database (2019)

Access to finance improve the survival rates, productivity and competence of MSEs. These enterprises in Debre-Markos town obtained from microfinance, *Iqub*, *Idir*, own capital and relatives than large banks (Tadesse, 2014). The main sources of initial capital for MSE’s are microfinance institution followed by bank and own capital (Alemu, 2018). Insufficient credit services for youth is a challenge in implementing rural youth economic development (Abdi, 2019). Financial institutions’ reluctance to give credit to young SMEs due to fear that firms may be defaulter (Nega & Hussein, 2016). The revolving funds of 10 billion birr for MSEs (FDRE, 2017) were not enough to ease financial challenges of the sector. The existence of inadequate loan size, borrowing cost and collateral requirement (Goshim & Tefera, 2018; Sissay, 2016; Tadesse, 2014), and high rate matching fund and liquidity problem for matching fund (Abeiy, 2017; Amentie et al., 2016; Sissay, 2016) constrained MSEs access to finance in Ethiopia. Moreover, loan duration affects MSEs access to finance from formal financial institutions (Petros, 2017; Tadesse, 2014). The business firms’ obstacle in Ethiopia (Fig. 4) showed that finance and electricity were the first and second major challenges in Ethiopia. As depicted in Fig. 5, finance is a major barrier and high loan rejection rate for MSEs than medium enterprises.

Micro and small enterprises (MSEs) needs business knowledge, skills and entrepreneurial orientation to profitably operate their business consistently in the existing business dynamics (Ghebremichael & Kassahun, 2014; Tarekegn et al., 2018). However, there are personal factor such as lack of business vision, risk averse of members, personal business exposure aggravate MSEs members dropout (Daba & Atnafu, 2016). The impact of aspiration to expand existing business and starting additional new business on growth of the MSEs is much higher for small enterprise compared to microenterprise (Amha, 2015).

#### Barriers against the development of MSMEs in Ethiopia

The existence of favorable working environment like government played a key role in the growth and development of MSEs (Hailu, 2016; Yimesgen, 2019). This support service program on average increased Dire Dawa MSEs monthly sales by 28%, employement by 42%, and capital asset formation by 60% (Eshetu et al., 2013). However, these supports are not sufficient for the development of micro and small enterprise (Hailu, 2016). In addtion, lack of training to start their own venture (Tewolde & Feleke, 2017), lack of awareness about the contribution and accessibility of consultancy service are the major problem of enterprises (Kidane et al., 2015). The ease of obtaining licenses to SMEs in Ethiopia was  better relative to sub-Saharan Africa region (Table 2).

**Table 2:** Number of days required to get license

	Obtain operating license		Obtain construction-related permit		Obtain import license	
	Small	Medium	Small	Medium	Small	Medium
Sub-Saharan Africa	17.7	17.9	42.5	49.1	14.8	15.2
Ethiopia	4.4	10.4	48	28.9	13.7	4.8

Source: authors calculation from World Bank enterprise survey (2015)

**Fig. 6:**
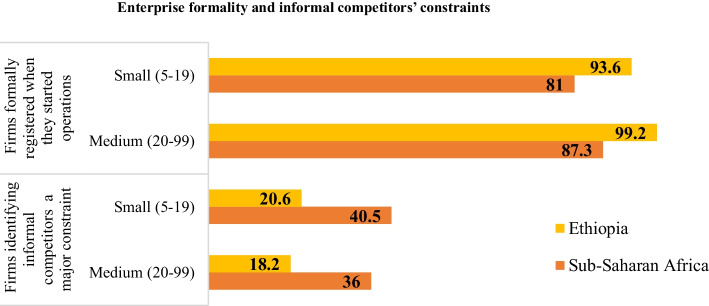
Informality of small and medium enterprise. Source: author calculation from World Bank Enterprise database (2020)

Micro and small enterprises were formally registered when they start operation in Ethiopia. The challenge of informal competitor lower in Ethiopia than sub-Saharan Africa and its effect decrease as firms grow from small to large enterprises (Fig. 6). This is due to large firms have the capacity to compete at large scale than small enterprise. Informal firms are also more credit constrained compared to formal firms (Aga & Reilly, 2011).

**Fig. 7:**
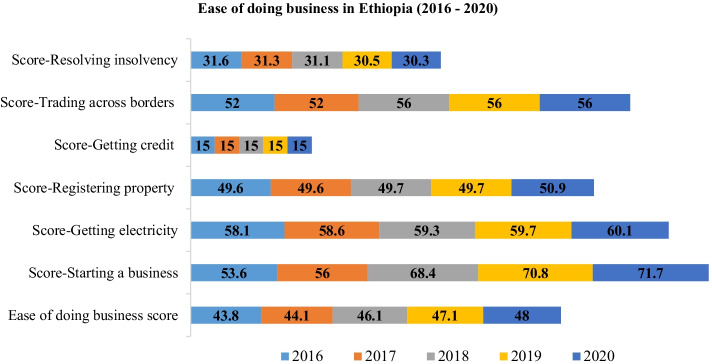
Performance of doing business in Ethiopia. Source: author calculation from World Bank Enterprise database (2020)

Micro and small enterprise access to sufficient premises in proper location increases enterprises financial performance (Ababiya, 2018). Poor infrastructure (Abeiy, 2017; Kinati et al., 2015) would cause more than 25% worktime loss daily due to power interruption (Cherkos et al., 2018) and business location identified as significant factors that hinder the growth of enterprises (Batisa, 2019). Power outages affected firms’ productivity, and the overall total cost due to outage increased by approximately 15% of firm’s aggregate cost (Abdisa, 2018). The cost of power outages for MSMEs in Addis Ababa is substantial, and a reduction of one power outage corresponds to a tariff increase of 16% (Carlsson et al., 2018). The location of enterprise effect on business performance raises two different arguments. Empirical evidences showed that MSEs desire to established in the center of town for attracting large customers even though rent in the downtown is high (Yimesgen, 2019). The second argument showed that MSEs that operate out of town have better performance. This is because MSEs have easy access for input and potential for business expansion (Kebeu, 2014). Entrepreneurial opportunities were increasing in Ethiopia, as presented in Fig. 7 over the 5 years. The score of ease of doing business increased over the last 5 years. However, the score of getting credit is stagnant which indicates access to finance were the long existing challenge of MSMEs development in Ethiopia.

The presence of market linkage enables MSEs to supply their produce and acquire inputs in the commercial value chain, which create jobs and improve efficiency of enterprises. However, the existing vertical linkage between MSEs and large enterprises are very limited, and limited access to raw materials (Mechalu, 2017; Mohammed & Beshir, 2019) and high cost of raw materials are major challenges of MSEs (Seifu, 2017). The absence of market linkage identified the critical problems of enterprises (Daba & Amanu, 2019; Dabi, 2017). Furthermore, there are weak institutional and sectoral linkages (Abera et al., 2019). As a result, informal linkages have a significant role to access market (Hadis & Ali, 2018).

## Conclusion

Micro, small and medium-sized enterprises (MSMEs) has been a key area of intervention to sustainable development specifically in growing youth population of sub-Saharan Africa. Given the implication of MSMEs in national development goals and it is a key development policy, there is little evidence particularly at broader context. Hence, this review article presents a systematic review of studies on the contribution of MSMEs in achieving sustainable development of Ethiopia and identifies the prevailing challenges. The paper has also demonstrated that MSMEs has myriad role in economy growth, poverty reduction, industrialization and livelihood as a whole. Micro enterprises in Ethiopia account the greatest share of employment from developing countries. Investing in small and medium-sized enterprises (SMEs) can contribute in some measure to 60% of the targets established in the SDGs. Manufacturing sector of Ethiopia micro-enterprises account for a staggering 97% of employment, 80% of employment growth in Tanzania accounted largely by informal enterprises. The review pointed that employment in micro-enterprises leveled as high in terms of its extent of importance to poverty reduction, empowered women socially, economically, and contributing to local economy and communities through income tax payment in Ethiopia.

The review revealed that lack of access to finance, poor infrastructure, and entrepreneurial attitudes are main challenges facing MSMEs in sub-Saharan Africa. Access to finance remains the largest obstacle for enterprises in the region. The problems became severe in time of crisis such as COVID-19 that lead two-thirds of micro and small firms in crisis and one-fifth of SMEs face risks of closing down permanently. The existence of inadequate loan size, borrowing cost and collateral requirement constrained MSEs in getting access to finance thereby the development of micro and small enterprise in Ethiopia. In addition, poor infrastructures are the main constraint that lead MSMEs to high worktime loss, reduce productivity, and increased cost of enterprise production.

## Recommendations and future research directions

Based on the review of studies, key implications for policy and future research include:

- It is essential to unlock entrepreneurship potential through integrated multi-sectoral and sustainable approach. Policy measures should prioritize inclusive financing schemes to vulnerable and marginalized entrepreneurs and enterprises that support business recovery during the crisis, and development of MSMEs.
- In addition, strong intervention to infrastructure development particularly electricity supply, working premise that increase ease of doing business and sustainable development of the country.
- Furthermore, this review calls for further research that focus on areas not given sufficient research attention such as the impact of MSMEs in achieving SDG 1 (No poverty), SDG 2 (Zero hunger), SDG 9 (Industrialization), SDG 12 (Responsible consumption and production). Future research needs to address ways to overcome the challenges hindering MSMEs’ development.

## Data Availability

Not applicable.

## Abbreviations

EEA: Ethiopia Economic Association
FMSEDA: Federal Micro and Small Enterprises Development Agency
GTP: Growth and Transformation Plan
MSEs: Micro and small enterprises
MSMEs: Micro, small and medium enterprises
NBE: National Bank of Ethiopia
NPC: National Plan Commission of Ethiopia
SDGs: Sustainable development goals
SMEs: Small and medium enterprises
